# Molecular detection of *Wolbachia* endosymbiont in reptiles and their ectoparasites

**DOI:** 10.1007/s00436-021-07237-1

**Published:** 2021-07-22

**Authors:** Ranju Ravindran Santhakumari Manoj, Maria Stefania Latrofa, Jairo Alfonso Mendoza-Roldan, Domenico Otranto

**Affiliations:** 1 Department of Veterinary Medicine, University of Bari, Valenzano, Italy; 2 Faculty of Veterinary Sciences, Bu-Ali Sina University, Hamedan, Iran

**Keywords:** *Wolbachia*, Lizard, *Ixodes ricinus*, *Neotrombicula autumnalis*, Snake

## Abstract

*Wolbachia*, a maternally transmitted Gram-negative endosymbiont of onchocercid nematodes and arthropods, has a role in the biology of their host; thus it has been exploited for the filariasis treatment in humans. To assess the presence and prevalence of this endosymbiont in reptiles and their ectoparasites, blood and tail tissue as well as ticks and mites collected from them were molecularly screened for *Wolbachia* DNA using two sets of primers targeting partial 16S rRNA and *Wolbachia* surface protein (*wsp*) genes. Positive samples were screened for the partial 12S rRNA and cytochrome *c* oxidase subunit 1 (*cox*1) genes for filarioids. Of the different species of lizards (*Podarcis siculus*,* Podarcis muralis* and *Lacerta bilineata*) and snakes (*Elaphe quatuorlineata* and *Boa constrictor constrictor*) screened from three collection sites, only *P. siculus* scored positive for *Wolbachia* 16S rRNA. Among ectoparasites collected from reptiles (*Ixodes ricinus* ticks and *Neotrombicula autumnalis*, *Ophionyssus sauracum* and *Ophionyssus natricis* mites), *I. ricinus* (*n* = 4; 2.8%; 95% CI, 0.9–7) from *P. siculus*, *N. autumnalis* (*n* = 2 each; 2.8%; 95% CI, 0.9–6.5) from *P. siculus* and *P. muralis* and *O. natricis* (*n* = 1; 14.3%; 95% CI, 0.7–55.4) from *Boa constrictor constrictor* scored positive for *Wolbachia* DNA. None of the positive *Wolbachia* samples scored positive for filarioids. This represents the first report of *Wolbachia* in reptilian hosts and their ectoparasites, which follows a single identification in the intestinal cells of a filarioid associated with a gecko. This data could contribute to better understand the reptile filarioid-*Wolbachia* association and to unveil the evolutionary pattern of *Wolbachia* in its filarial host.

## Introduction

*Wolbachia*, an obligate intracellular endosymbiont of arthropods and filarioids, has gained more attention in the scientific community due to its role in the biology of filarioids and their vectors and its contribution in the development of immunopathology of filariasis (Bandi et al. 1998; Taylor, 2003). *Wolbachia pipientis* represent the single species of the genus *Wolbachia* with supergroups used to classify the large genetic diversity of different strains identified (Casiraghi et al. 2005). This endosymbiont has been reported in around 50% of the terrestrial arthropod species which includes insects, mites, crustaceans, spiders, scorpions, collembolans and in several species of onchocercid nematodes (Ferri et al. 2011; Lefoulon et al. 2016; Manoj et al. 2021), as well as in non-filarial plant nematodes of the order Tylenchida (Haegeman et al. 2009). Onchocercid-*Wolbachia* association is obligatory being required for the reproduction, development and long-term survival of the host (Martin and Gavotte 2010). Meanwhile, arthropod-*Wolbachia* association could be considered more parasitic, since the bacteria gain fitness advantage by reproductive manipulations of the host, modifying the iron homeostasis and reducing the vector capacity of their hosts (Bandi et al. 2001; Kremer et al. 2009; Martinez et al. 2017). Based on their genetic evolution, *Wolbachia* have been classified as 17 supergroups, from A to S (Fenn et al. 2006; Lefoulon et al. 2020a, b), in which *Wolbachia* of arthropods were included in supergroups A, B, E, H, I, K, that of nematodes in C, D, J (Glowska et al. 2015) and plant-parasitic nematodes in L (Brown et al. 2016). Among these supergroups, F is an exception being reported in both arthropods (i.e. termites, spiders, mites, bugs; Rasgon and Scott 2004; Ros et al. 2009) and onchocercid nematodes (i.e., human filariae, *Mansonella*; filariae of black bear, *Cercopithifilaria japonica*; filariae of geckoes, *Madathamugadia hiepei*; Casiraghi et al. 2001; Casiraghi et al. 2005; Keiser et al. 2008; Ferri et al. 2011; Lefoulon et al. 2012). For long time, *Wolbachia* was considered to be present only in two of the eight onchocercid subfamilies, i.e. Onchocercinae and Dirofilariinae (Lefoulon et al. 2012). However, the discovery of this bacterium in the filariae of geckoes (i.e. Splendidofilariinae) suggested that *Wolbachia* diverged before the first bacterial invasion in onchocercid lineage (Bandi et al. 1998; Fenn et al. 2006; Casiraghi et al. 2004). Moreover, previous inferences about the existence of a congruency between *Wolbachia* and the filarial host phylogenies (Bandi et al. 1998) has become inaccurate after the addition of supergroup F (Lefoulon et al. 2012). Though *Wolbachia* was detected once in filarial worms from geckoes (i.e., *M. hiepei*), its presence in the reptilian species is not known. Hence the current study aimed to assess the presence and prevalence of *Wolbachia* in synanthropic lizards and snakes and their ectoparasites from southern Italy.

## Materials and methods

### Sample collection

The reptiles and ectoparasites collected under the frame of previous studies (Mendoza-Roldan et al. 2019, 2021a) were included in the study. Specimens were collected from two areas of the Apulia region (site 1, a peri-urban region in Bari and site 2, a zoological park in Fasano), and from Basilicata region (site 3) in South Italy. From site 1, 38 mite infested lizards, and from site 3, 128 lizards and snakes were captured (Mendoza-Roldan et al. 2019). Reptile species were identified by using specific reference keys (Arnold, 2002). Blood samples were collected in EDTA vials via tail fracture/cardiocentesis and from the ventral coccygeal vein in case of lizards and snakes, respectively, and stored at − 20 °C until further processing. A small portion of the tail tissue was also collected from lizards and stored in 70% ethanol until DNA isolation.

### DNA isolation, molecular and sequencing analyses

Genomic DNA from blood of reptiles and tail tissues of lizards were extracted using Qiagen blood Mini kit and Qiagen DNeasy Blood & Tissue kit (Qiagen, Hilden, Germany), respectively, following the manufacturer’s instructions, whilst guanidine isothiocyanate protocol (GT) (Chomkzynski, 1993) was used to extract the DNA from ticks and mites, and the elutions were made in 50 μl AE buffer. All samples were tested molecularly for the endosymbiont, *Wolbachia* using two sets of primers targeting partial 16S rRNA and *Wolbachia* surface protein (*wsp*) genes and the positive samples were screened for the partial 12S rRNA and cytochrome *c* oxidase subunit 1 (*cox*1) genes for filarioids (Casiraghi et al. 2004; Martin et al. 2005; Otranto et al. 2011; Zhou et al. 1998). All amplified PCR products were visualized in 2% agarose gel containing Gel Red® nucleic acid gel stain (VWR International PBI, Milan, Italy) and documented in Gel Logic 100 (Kodak, NY, USA). The PCR products were purified and sequenced in both directions using the same primers, employing the Big Dye Terminator v.3.1 chemistry in a 3130 Genetic analyzer (Applied Biosystems, CA, USA) in an automated sequencer (ABI-PRISM 377). Nucleotide sequences were edited aligned and analysed using BioEdit and compared with available sequences in the GenBank using Basic Local Alignment Search Tool (BLAST; http://blast.ncbi.nlm.nih.gov/Blast.cgi).

### Phylogenetic analyses

Representative sequences of the 16S rRNA of *Wolbachia* were included along with the sequences available in the GenBank database for phylogenetic analyses. Phylogenetic relationships were inferred using maximum likelihood (ML) method based on Kimura 2-parameter model (Kimura, 1980) with discrete Gamma distribution (+ *G*) to model evolutionary rate differences among sites selected by best-fit model (Kumar et al. 2018). Evolutionary analyses were conducted on 1000 bootstrap replications using the MEGA X software (Tamura et al. 2013). Homologous sequences from *Ehrlichia ruminantium* and *Ehrlichia chaffeensis* were used as outgroups (NR074513, AF147752).

### Statistical analysis

Prevalence of *Wolbachia* infection among reptiles (proportion of reptiles and ectoparasites infected by *Wolbachia* on the total population of reptiles and ectoparasites collected from different sites) was assessed. Statistical analysis was done using StatLib software. Exact binomial 95% confidence intervals (CI) were established for proportions.

## Results

**Fig. 1:**
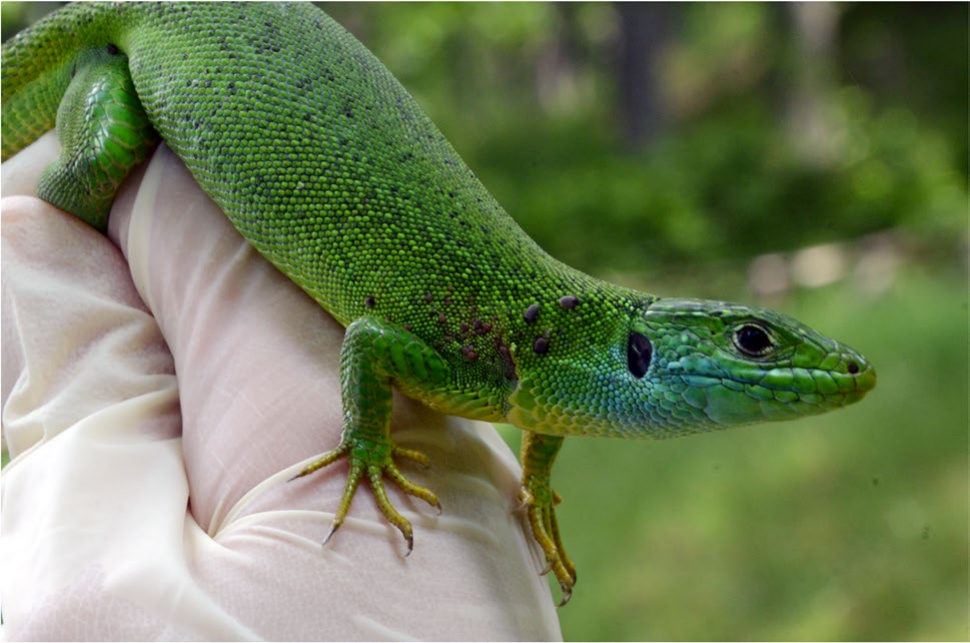
*Lacerta bilineata* infested with *Ixodes ricinus*

**Fig. 2:**
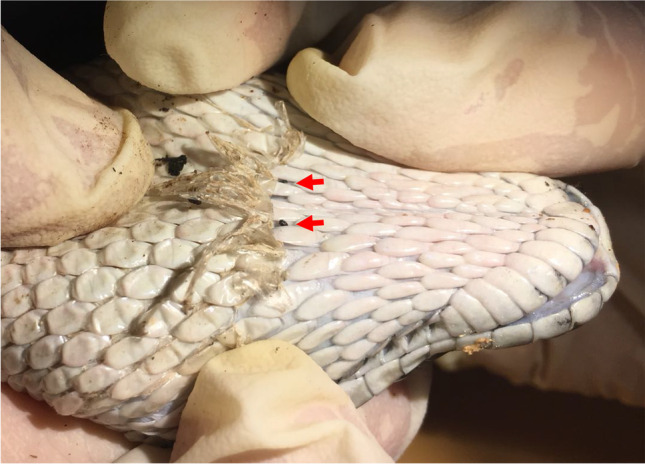
*Boa constrictor constrictor* infested with *Ophionyssus natricis* (red arrows)

Of the different species of lizards (*Podarcis siculus*,* Podarcis muralis* and *Lacerta bilineata*) and snakes (*Elaphe quatuorlineata* and *Boa constrictor constrictor*), only the blood (n = 5, 3.4%; 95% CI, 1.37–7.8) and tissue samples (n = 3, 2.1%; 95% CI, 0.6–6.1) of *P. siculus* scored positive for *Wolbachia* 16S rRNA. Of the 5 *Wolbachia* positive blood samples, 2 were collected from site 1 and 3 from site 3, whilst all positive tissue samples were collected from site 3. Among ectoparasites collected from reptiles (*Ixodes ricinus* tick and *Neotrombicula autumnalis*, *Ophionyssus sauracum* and *Ophionyssus natricis* mites) (Figs. 1, 2), *I. ricinus* (n = 4; 2.8%; 95% CI, 0.9–7) from *P. siculus*, *N. autumnalis* (n = 2 each; 2.8%; 95% CI, 0.9–6.5) from *P. siculus* and *P. muralis* and *O. natricis* (n = 1; 14.3%; 95% CI, 0.7–55.4) from *Boa constrictor constrictor*, scored positive for *Wolbachia.* In particular, positive ticks were collected from both site 1 (n = 1, 2.6%; 95% CI, 0.1–13.3) and 3 (n = 3, 2.5%; 95% CI, 0.7–7.4), while positive mites were collected from sites 2 (n = 1, 33.3%; 95% CI, 1.7–86.5) and 3 (n = 4, 3.4%; 95% CI, 1.2–8.3). The lizard and ectoparasite samples collected from site 2 had the highest prevalence (33.3%) of *Wolbachia* followed by site 3 (5.9%) and site 1 (2.3%). Corresponding reptilian hosts of the ectoparasites positive for *Wolbachia* DNA were negative for this endosymbiont. PCR products of *wsp* gene had non-specific amplifications, whereas none of 16S rRNA positive *Wolbachia* samples scored positive for filarioids*.*

**Fig. 3:**
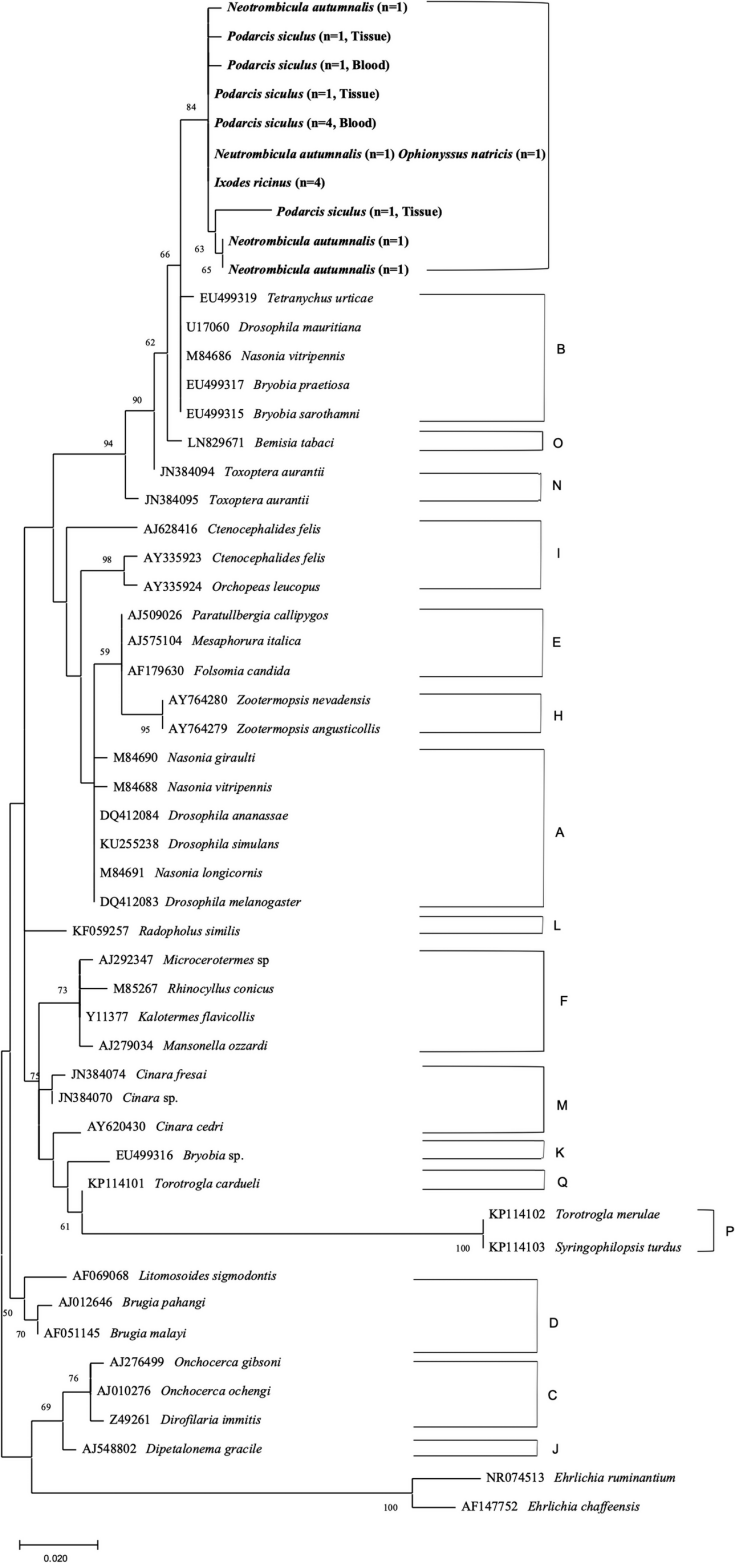
Phylogenetic relationship of *Wolbachia* detected in this study (in bold) and other available from GenBank belonging to different supergroups based on a partial sequence of the 16S rRNA gene. Evolutionary analysis was conducted on 1000 bootstrap replications using Maximum Likelihood method and Kimura 2-parameter model with discrete Gamma distribution (+ *G*) to model evolutionary rate differences among sites selected by best-fit model. GenBank accession number and host species are indicated

Phylogenetic analysis of the 16S rRNA gene yielded strict consensus in overlapping the topology of the trees, with a strong bootstrap value (up to 100%) for each supergroup clade (Fig. 3). All sequences of *Wolbachia* detected in lizard and reptile ectoparasites were clustered in two monophyletic clades (bootstrap value of 84%) and as sister clade of that of *Wolbachia* supergroup B. Representative sequences of *Wolbachia* sequences were deposited in GenBank (accession numbers: MZ490470-MZ490476).

## Discussion

The current study assessed the presence of *Wolbachia* in tail tissues and blood of lizards suggesting its circulation in these host species.

Though the presence of this bacterium has been previously reported in the intestinal cells of the saurian filarioid *M. hiepei* (Lefoulon et al. 2012), it has never been identified in reptilian hosts and/or their ectoparasites. In filarioids this endosymbiont is usually located in hypodermal lateral chords (Taylor and Hoerauf 1999; Kramer et al. 2003). Previous studies suggested that the release of *Wolbachia* into the blood occurs following the death or damage of the worms after anti-filarial chemotherapy (Taylor 2003). In the current study, though the presence of this bacterium was detected in the reptilian host, none of them were positive for any filarioid. This suggests that the associated filarioids and their larval stages may be located in tissues other than tail or blood. Filarial nematodes belonging to the genera *Foleyella*, *Macdonaldius*, *Splendinofilaria* and *Oswaldofilaria* are found to infect reptiles vectored by mosquitoes (Mendoza-Roldan et al. 2021b). Most of them are found in extra intestinal sites such as the lungs, circulatory system and subcutaneous tissue (Jacobson et al. 2020). It has been also postulated that the presence of blood circulating *Wolbachia* antigens might result from the natural excretion of *Wolbachia* products by the nematodes or the release of these products from the dying worms (Shiny et al. 2009; Anuradha et al. 2012). Similarly, immunohistology studies in human onchosarcoma and blood cells have suggested free endobacteria outside of the filariae in host’s tissues (Brattig et al. 2004) which further strengthens the possibility of finding the *Wolbachia* in host blood and tissues. Indeed, the finding of *Wolbachia* in reptiles opens the possibility of widening the knowledge about the evolution of this endobacterium.

The presence of *Wolbachia* in mites of the species *N. autumnalis* and *O. natricis* is new to the scientific community and could be of importance considering the biodiversity in trombiculid and macronyssid mites from reptiles (Mendoza-Roldan et al. 2020; Bezerra-Santos et al. 2021). In trombiculid mite species, only larval stages are parasitic and the infestations are self-limiting (Bassini-Silva et al. 2017; Jacinavicius et al. 2018), which reduces the possibility of translocation of the mite from an infected to an uninfected host. The finding of *Wolbachia* positive mites from the endosymbiont negative reptiles may indicate a vertical transmission of this bacterium. Conversely, it was suggested that presence of *Wolbachia* in the hypodermal cells of onchocercid nematodes is an ancestral somatic tissue-preference, which may aid the horizontal transmission (Landmann et al. 2012; Lefoulon et al. 2012). The latter transmission pattern could also be explained by *Wolbachia* natural excretion or as a consequence of dying filarial worms releasing endobacteria in host’s tissues and blood (Brattig et al. 2004; Shiny et al. 2009; Anuradha et al. 2012). The finding of infected *I. ricinus* on uninfected host can be explained by the translocation of this ectoparasite while feeding (i.e. from an infected host to an uninfected host). However, the natural occurrence of this bacterium in ticks and mites cannot be ruled out, given the high prevalence (i.e. from 20 to around 75%) reported amongst arthropods (Werren et al. 1995; Jeyaprakash and Hoy 2000). Phylogenetic relationship of *Wolbachia* from reptiles and its ectoparasites with supergroup B suggests the possibility of a horizontal transfer of this bacterium from the host to the ectoparasites or vice versa. Moreover, the low genetic divergence in supergroup F when compared to supergroups C and D and a close resemblance with supergroups B and A also supports the hypothesis that horizontal transmission has occurred between Onchocercidae and arthropods (Lefoulon et al. 2012).

## Conclusion

Being firstly reported from the reptilian host and their ectoparasites, a detailed investigation is needed to understand the relationship of this reptilian *Wolbachia* and the associated filarial host. The new findings may further complement the evolutionary picture of *Wolbachia* and its filarial host. Hence, detailed research in this area will help to understand the tropism of this endosymbiont in its reptilian filarioid host and to unravel the evolutionary traits of this enigmatic bacterium.
